# Dexamethasone-Loaded Lipomers: Development, Characterization, and Skin Biodistribution Studies

**DOI:** 10.3390/pharmaceutics13040533

**Published:** 2021-04-11

**Authors:** Eloy Pena-Rodríguez, Maria Lajarin-Reinares, Aida Mata-Ventosa, Sandra Pérez-Torras, Francisco Fernández-Campos

**Affiliations:** 1 Topical & Oral Development R+D Reig Jofre Laboratories, 08970 Barcelona, Spain; eloy.pena@reigjofre.com (E.P.-R.); mlajarin@reigjofre.com (M.L.-R.); 2 Molecular Pharmacology and Experimental Therapeutics, Department of Biochemistry and Molecular Biomedicine, Institute of Biomedicine (IBUB), University of Barcelona, 08028 Barcelona, Spain; aidamatav@ub.edu (A.M.-V.); s.perez-torras@ub.edu (S.P.-T.); 3 Biomedical Research Networking Center in Hepatic and Digestive Diseases (CIBEREHD), Carlos III Health Institute, 28029 Madrid, Spain; 4 Sant Joan de Déu Research Institute (IR SJD-CERCA) Esplugues de Llobregat, 08950 Barcelona, Spain

**Keywords:** follicular targeting, dexamethasone, alopecia areata, lipomers, lipid polymer hybrid nanocapsules, biodistribution, skin, ethyl cellulose

## Abstract

Follicular targeting has gained more attention in recent decades, due to the possibility of obtaining a depot effect in topical administration and its potential as a tool to treat hair follicle-related diseases. Lipid core ethyl cellulose lipomers were developed and optimized, following which characterization of their physicochemical properties was carried out. Dexamethasone was encapsulated in the lipomers (size, 115 nm; polydispersity, 0.24; zeta-potential (Z-potential), +30 mV) and their in vitro release profiles against dexamethasone in solution were investigated by vertical diffusion Franz cells. The skin biodistribution of the fluorescent-loaded lipomers was observed using confocal microscopy, demonstrating the accumulation of both lipomers and fluorochromes in the hair follicles of pig skin. To confirm this fact, immunofluorescence of the dexamethasone-loaded lipomers was carried out in pig hair follicles. The anti-inflammatory (via TNFα) efficacy of the dexamethasone-loaded lipomers was demonstrated in vitro in an HEK001 human keratinocytes cell culture and the in vitro cytotoxicity of the nanoformulation was investigated.

## 1. Introduction

Nanoparticles are pharmaceutical forms that are used to improve the efficacy and bioavailability of different poorly water-soluble active ingredients. The physicochemical properties of nanoparticles, such as their high surface-to-volume ratio, the ability to increase permeability through different cell tissues, the possibility of increasing the solubility of active ingredients, or the ability to modulate the release kinetics to achieve depot formulations, make nanoparticles a very attractive approach to improve the therapeutical indices of active ingredients through different routes of administration.

In recent decades, there has been a great deal of research related to modulated targeting and localized release in different parts of the body. The stratum corneum, comprised of a group of low water content corneocytes and a lipid matrix made of ceramides and cholesterol as the main components, acts as a barrier for topical products in a very efficient way [1]. In the past few years, different nanoencapsulation platforms have been developed to transport drugs to the inner layers of the skin. Regarding the transdermal route, it is well-known that flexible particles, such as transfersomes, or micellar systems are capable of increasing permeation [2,3]; however, the loading capacity of these systems is usually low. In recent years, the transfollicular route has gained more attention, as it has been suggested to increase dermal absorption, probably due to accumulation in the pilosebaceous unit. For example, Lademann et al. demonstrated that, by using nanoparticles made of poly (lactic-co-glycolic acid) (PLGA), it was possible to obtain a depot effect for 10 days in porcine hair follicles, whereas nanoparticles on the skin surface were only detectable for 24 h [4]. Furthermore, nanosystems, such as polymeric nanoparticles or nanostructured lipid carriers, have higher loading capacities [5]. However, there is still a lack of information regarding the accumulation of these systems in the anatomy of the hair follicle.

Lipomers, or lipid core polymeric nanocapsules (LPNCs), are oily vesicles surrounded by a polymeric wall [6]. In polymeric nanospheres, the active is trapped both on the surface of the particle and in its core. They usually have a burst release effect, due to the desorption of the active from the surface, followed by a plateau based on diffusion/erosion mechanisms. Urzula Bazylinska et al. compared the release of a lipophilic active encapsulated in nanospheres and lipid polymer hybrid nanocapsules and verified that the presence of the lipid nucleus increased the encapsulation efficiency and decreased the burst release effect, obtaining a more sustained release [7].

Topical corticosteroids are the most frequently used drugs for the treatment of inflammatory diseases. Dexamethasone (DEX) has been used topically to treat ocular inflammation [8], atopic dermatitis [9], and alopecia areata (AA) [10], among other diseases, with a high benefit-to-risk ratio. In the case of AA, topical corticoids are frequently ineffective and oral or intralesional therapy is required; furthermore, the potential adverse effects and patient discomfort caused by scalp injections must be considered [11]. Some authors have used solutions with a high proportion of ethanol (70%), as a permeation enhancer, to deliver DEX [9]. They found drug accumulation exclusively in the lipid interspaces and did not observe efficient penetration into corneocytes. There exist other topical corticoids in the market, such as mometasone or methylprednisolone, which are also formulated in hydroalcoholic vehicles with a high content of ethanol. Ethanol has been associated with skin irritation or contact dermatitis [12], it could alter the stratum corneum structure [13], and modify the skin microbiota [14], which could be related to skin diseases and inflammatory status. Thus, it is preferable to minimize its use in topical formulations. Other authors encapsulated DEX (hemisuccinate) in liposomes by microfluidics and film hydration techniques [15]. Even though liposomes are very attractive drug delivery systems, the loading capacity is usually low and could be an issue for specific indications that require higher doses. Considering the above, the improvement of topical treatment is still required, to increase patient quality of life and to minimize adverse effects. Lipomers are a promising drug delivery system to improve the therapeutical balance of DEX in AA treatment, due to possible accumulation in the pilosebaceous unit, where the disease takes place, and under the hypothesis that the lipid nucleus increases the loading capacity. This fact leads to a higher drug concentration in the LPNCs, compared with similar particles without the lipidic core, for which the DEX loading capacity was about 1–2% [16]. 

For this study, DEX-LPNCs were developed and characterized, in terms of their physicochemical properties and drug release. A description of the skin biodistribution and its accumulation on hair follicles is given. Additionally, information about the associated in vitro cytotoxicity and efficacy is shown. It was found that the proposed DEX-loaded LPNCs have potential as a tool for the topical treatment of AA and other inflammatory skin diseases.

## 2. Materials and Methods

### 2.1. Materials

DEX (Fagron Ibérica, Barcelona, Spain), ethyl cellulose (EC) (Ashaland Industries Europe GmbH, Rheinweg, Switzerland), Tween 80 and Span 60 (Croda Iberica S.A., Spain), benzalkonium chloride (Sigma Aldrich, Madrid, Spain), medium chain triglycerides (MCT) (Oxi-Med Expres S.A., Barcelona, Spain), ethanol absolute (ET) and ethyl acetate (EA) (Scharlab S.L., Barcelona, Spain), and purified water (Inhouse) were used to formulate the LPNCs. Coumarin 6 (C6) (Sigma Aldrich, St. Louis, MO, USA) and 1,2-dioleoyl-3-[16-N-(lissamine rhodamine B sulfonyl) amino]palmitoyl-sn-glycerol (LRB) (Avanti Polar Lipids, Alabaster, AL, USA) were used as fluorochromes. Paraformaldehyde (Scharlab S.L., Barcelona, Spain), Sucrose (Acor, Valladolid, Spain), optimal cutting temperature compound (OCT) (Tissue-Tek Sakura Finetek, Torrance, CA, USA), rabbit anti-DEX IgG (Abcam, Cambridge, UK), Alexa Fluor 488 goat anti rabbit (Life Technologies, Carlsbad, CA, USA), Prolong^TM^ Gold Antifade mounting medium (Thermo Fisher Scientific, Barcelona, Spain), and Tween 20 (Sigma-Aldrich, St. Louis, MO, USA) were used to prepare samples for microscopic visualization.

### 2.2. Synthesis of DEX-Loaded LPNCs, Fluorescent-Loaded LPNCs, and Non-Vesiculated Control Solutions

LPNCs were produced by the Emulsion Solvent Evaporation method invented by Fessi et al. [17]. In this method, an organic phase composed of different ratios of EA and ET, EC (2.33%, *w*/*w*), MCT (0.2%, *w*/*w*), DEX (1%, *w*/*w*), and different concentrations of Span 60, were emulsified in an aqueous phase composed of different concentrations of Tween 80 and benzalkonium chloride (preservative). Emulsification was carried out under probe sonication (amplitude of 40%, 5 min; energy, 7000 W; frequency 23.88 kHz) using a UP400st ultrasonic device (Hielscher Ultrasonics, Germany), to reduce the size of the emulsion droplet. After formation of the emulsion, the system evaporated the organic solvents under vacuum in a rotary evaporator, which led to polymer precipitation and lipomer formation. The whole process was performed at room temperature.

A solution of DEX was prepared in a 1% hydroethanolic solution (90:10 *v*/*v*, water:ethanol) for use as a non-encapsulated DEX control (FREE-DEX). LRB- and C6-loaded LPNCs (LRB-C6-LPNCs) were manufactured with the same composition as previously described, but without DEX. LRB was added to the lipid nucleus in a mole-to-mole ratio of MCT:LRB of 1:1000, according to Lymberopoulos et al. [18]. In addition, 0.1% *w*/*w* C6 was incorporated into the organic phase (EA:ET), simulating the encapsulation of a hydrophobic active ingredient. Fluorochrome control aqueous solutions used in confocal microscopy were prepared with 3% Tween 80 of LRB and C6, at the same concentration as in the LRB-C6-LPNCs, for use as a control for non-vehicle fluorophores (FREE-LRB-C6).

### 2.3. Screening of Experimental Variables on Nanoparticle Properties

The properties of the lipomers are influenced by different experimental variables, such as the amount of ingredients and manufacturing conditions. Some preliminary screening trials were performed. 

Different batches were produced to fix the % *w*/*w* of EC and MCT, in order to maximize the loading capacity of DEX in the LPNCs, while maintaining the hydrodynamic diameter below 150 nm and the polydispersity below 0.3. To select the organic solvent, the viscosity of EC was characterized (Brookfield RDV-III Ultra, Spain. Spindler: SC4-21, speed: 100 rpm; temperature: 25 °C) in EA:ET (1:0, 0:1, 1:1, 1:5, and 5:1 *v*/*v*) and the mixture with the lowest viscosity was selected.

The samples were subjected to rotary evaporation in a Heidolph VV1 rotary evaporator (Heidolph Instruments, Germany) with a thermostatized bath at 40 °C for 5, 7, 10, and 15 min. The residual concentration of EA and ET was analyzed by gas chromatography (Agilent A7890 with Headspace AG1888, Santa Clara, CA, USA) following European Pharmacopoeia 7.0, chapter 2.4.24 (Identification and control of residual solvents) [19]. Briefly, carrier gas (Helium) flowed at 1.5 mL/min in a column (DB-WAX, 60 m, 0.25 mm, 0.25 µm; Agilent Technologies, USA) for 30 min. The split ratio was 20, the injector Temperature (Tª) was 250 °C, and the detector Tª was 270 °C. The Tª ramp was increased by 10 °C/min.

In order to obtain stable nanoformulations against aggregation, the influences of the surfactant Tween 80 and co-surfactant Span 60 (Table 1) on the Z-potential (mV), nanoparticle hydrodynamic diameter or Z-average (nm), polydispersity index (PDI), and encapsulation efficiency (%EE) were studied. Statistical analysis of the variables studied were carried out using Minitab 17 statistical software (Minitab, Inc., 2010, State College, PA, USA), with the significance level set as α = 0.05.

### 2.4. LPNCs Physic-Chemical Characterization

The hydrodynamic size, polydispersity index (PDI), and Z-potential were studied for the LPNCs through Dynamic Light Scattering (DLS) using a Malvern Zetasizer Nano ZS (Malvern Panalytical, Malvern, UK). A dilution of 1:10 in milliQ water was performed to adjust the intensity (attenuator position between 3 and 6).

The LPNCs’ morphology, size, and distribution were studied using Transmission Electron Microscopy (TEM). The LPNCs’ size was also measured using the particle analysis tool of the Image J software. The TEM grids were coated with formvar of a 1:10 dilution of LPNCs in milliQ water and incubated for 1 min at room temperature. The grids were washed with milliQ water and stained with a 2% *w*/*w* uranyl acetate solution (Electron Microscopy Sciences, Hatfield, England) during 1 min at room temperature. They were then dried in a petri dish overnight and observed using a TEM microscope (Jeol JEM 1010 100 kv; Jeol, Tokyo, Japan).

### 2.5. DEX Quantification

DEX was quantified through HPLC, in terms of encapsulation efficiency (*%EE*), according to an indirect method (Equation (1)):

(1) $$\%EE=\frac{W_{T}-W_{NE}}{W_{T}}\times \;100,$$

where *W_NE_* is the amount of DEX quantified in the filtrate (DEX not encapsulated) and W_T_ is the DEX quantified in the total formula. The LPNCs were centrifuged in 100 KDa amicon ultra (Merck Millipore, Barcelona, Spain) at 4500 rpm for 30 min. DEX in the filtrate and in LPNCs (total drug content) were analyzed using a High-Performance Liquid Chromatograph (HPLC) Alliance, with a photodiode array detector (PDA). A C18 column (250 × 4.6 mm, 3 µm) was used with an isocratic mixture of acetonitrile:KH_2_PO_4_ 0.05 M buffer (60:40) as the mobile phase, with a flow rate of 1.8 mL/min and an injection volume of 20 µL. The sample and column temperatures were 25 °C.

The amount of total drug entrapped per weight of nanoparticle or loading capacity (*%LC*) was calculated using the following equation:

(2) $$\%LC=\%EE\;\times \;\frac{M_{DEX}}{M_{LPNCs}},$$

where *M_DEX_* is the amount of active ingredient initially added to the formulation and *M_LPNC_*_s_ is the mass of the nanoparticle components.

### 2.6. In Vitro Release Tests of the DEX-Loaded LPNCs

In vitro release of DEX from the LPNCs was studied using vertical Franz cells (Vidrafoc, Barcelona, Spain) with a 12 mL receptor compartment and an effective diffusion area of 1.54 cm^2^. A mixture of ethanol and purified water (50:50) was used as receptor medium (RM), at 32 °C and stirred at 500 rpm, to keep the sink conditions throughout the experiment. A total of 0.6 g of LPNCs was applied in the donor compartment, corresponding to 60 mg of DEX. The membrane used was a dialysis membrane (Spectrum Chemical, New Brunswick, NJ, USA) with pore diameter of 12–14 kDa.

Aliquots of 300 µL were taken at certain times (1, 2, 3, 4, 5, 21, 22, 23, and 24 h) and injected into the HPLC with the method described in Section 2.5, to quantify the amount of DEX that had diffused through the membrane.

Kinetic modelling of the release data was studied with the DD-solver [20] Excel Add-on, using a non-linear approach. Model selection was based on the lowest Akaike Information Criteria (AIC), reflecting the lowest deviation of the model with respect to the empirical data [21]. Firstly, the mean release values were adjusted to the model described in Table 2, to obtain the mean population behavior. Then, individual data were adjusted, according to the model selected. The mean and standard deviation of the parameters were reported.

In Table 2, F is the fraction of active released at time *t*, *F_max_* is the maximum fraction of active released (i.e., at infinite time), K_1_ is the first-order constant, *K_H_* is the Higuchi constant, *K_KP_* is the Korsmeyer–Peppas constant (related to the structural and geometric character of the drug release matrix), *n* is the diffusional exponent indicating the drug-release mechanism (if *n* is less than 0.43, then a Fickian diffusion release mechanism is implied; if *n* is between 0.43 and 0.85, then the release mechanism follows an anomalous transport mechanism), T_d_ represents the time at which 63.2% of the drug is released, and β is the Weibull shape parameter. For values of β lower than 0.75, the release follows a Fickian diffusion, either in Euclidian (0.69 < β < 0.75) or fractal (β < 0.69) space. Values of β in the range of 0.75–1.0 indicate a combined mechanism, which is frequently encountered in release studies [22,23].

### 2.7. Pig Skin Permeation 

Pig skin was obtained from a local abattoir (Barcelona, Spain) at the moment of sacrifice. Full thickness skin pieces were defatted (with a scalpel) and frozen at −20 °C until use. On the day of the experiment, skin pieces were mounted on Franz cells with an effective diffusion area of 0.196 cm^2^ and around 12 mL of receptor volume capacity (PBS at 32 °C and stirred at 500 rpm). TEWL measurements were performed, to check the integrity of the skin samples [12] (TEWL Vapometer SWL4549, Delfin Technologies Ltd., Kuopio, Finland). A solution dose of 76 mg of each of the formulations were given in infinite dose in non-occluded conditions.

#### 2.7.1. Confocal Microscopy Biodistribution of Fluorescent Probes

To study the biodistribution of LPNCs in the skin, confocal fluorescence microscopy was used. For this, C6 was encapsulated in the nanoparticles, to simulate the biodistribution of a hydrophobic small molecule, and LRB was used as fluorophore covalently bound to a lipid (palmitic acid) and included in the lipid core of the LPNCs, in order to track the nanoparticles. The labelled LRB-C6-LPNCs were purified using Visking dialysis tubing with a cut-off of 12,000–14,000 Dalton. The non-encapsulated fraction of both fluorophores was quantified by spectrophotometry (VICTOR Multilabel Plate Reader, Perkinelmer, Massachusetts, USA) and the *%EE* was calculated following Equation (1).

Full thickness pieces of pig skin were warmed and placed in Franz cells, according to Section 2.7. After 18 h of permeation, the diffusion surface was washed with PBS, cut with a scalpel into pieces of about 0.5 cm^2^, and fixed in 4% *w*/*w* paraformaldehyde solution for 5 min. Then, the skin samples were incubated in aqueous solutions of increasing sucrose concentration (5%, 15%, and 25% *w*/*w*) for 15 min in each solution. They were then placed in plastic molds and dipped in OCT to cut on a cryostat Leica CM 3050 S (Leica Biosystems, Barcelona, Spain) to a 50 µm thickness. The slices were collected on poly-lysine-coated slides and washed with PBS and 0.05% Tween 20 (TPBS) for 5 min, to remove the OCT and permeabilize the samples. On the day of observation, sections were incubated with 15 µL of Hoescht solution (2 µg/mL) for 10 min and washed with TPBS to stain the cell nuclei. The samples were analyzed under a confocal microscope (Leica Microsystems, Wetzlar, Germany). The emission laser wavelengths were 570, 500, and 525 nm and the excitation wavelengths were 561, 488, and 405 nm for LRB, C6, and Hoescht, respectively. About 20 planes were obtained per image, separated by a 3 µm step. Composites of the different planes were created, in terms of the brightest point for each pixel, through the ImageJ tool Z-stack (ImageJ2 v2.35, National Institutes of Health, Bethesda, MD, USA). A skin blank was processed in the same way as the test samples, to quantify skin autofluorescence. The mean intensity was measured with the ImageJ software and was subtracted from the intensity of the red and green channels of the samples.

#### 2.7.2. Immunohistofluorescence Biodistribution

To study the biodistribution of the DEX carried in the LPNCs, immunohistofluorescence (IHF) experiments were carried out. Permeation and skin slices were carried out in the same way as in Section 2.7.1, but the slices were 10 µm thick. Then, 15 µL of a 1/300 dilution of the rabbit polyclonal primary anti-DEX IgG were added, and the samples were left to incubate in a humid environment overnight at 4 °C. Then, the samples were washed with TPBS and incubated with a 1/300 diluted fluorescent secondary goat anti-rabbit IgG Alexa 488 for 2 h under the same incubation conditions. Finally, ProLong Gold Antifade mounting medium was added. The samples were analyzed under a fluorescent field effect microscope. A L5 Leica filter cube was used, and the filters were BP480/40, BS505 for excitation, and BP 527/30 for emission. To study the possible non-specific interactions of the skin, the same process was carried out with non-treated skin samples and processed in the same way as test samples. The resultant intensity was subtracted from the profiles of the samples using the ImageJ software.

### 2.8. In Vitro Cytotoxicity/Anti-TNFα Efficacy

Cell culture: The HEK001 cell line was obtained from ATCC (Promochem Partnership, Manassas, VA, USA). HEK001 was grown in monolayer on a solid support, at 37 °C in a humidified atmosphere with 5% CO_2_ and maintained in keratinocyte serum-free media supplemented with 100 µg/mL epidermal growth factor (EGF) (Life Technologies, Carlsbad, CA, USA), 20 U/mL penicillin, and 20 μg/mL streptomycin (Life Technologies). Mycoplasma-free maintenance was confirmed every two weeks by PCR amplification.

Cell treatments: For the cell viability assays, HEK001 cells were seeded at a density of 5000 cells/well in 96-well culture plates. At 24 h post-seeding, cells were treated with increasing doses of DEX-charged LPNCs, non-charged LPNCs, or the corresponding amount of benzalkonium chloride for 24 h. After 48 h, cell viability was assessed using an MTT (3-[4,5-dimethylthiazol-2-yl]-2,5 diphenyl tetrazolium bromide) colorimetric assay (Sigma-Aldrich, St. Louis, MO, USA). Cell survival for all experiments was expressed as the percentage of viable cells, relative to that in untreated cells (defined as 100%). For the cytokine modulation assays, HEK001 cells were seeded at a density of 700,000 cells/well in 6-well culture plates. At 24 h post-seeding, cells were pre-treated with 10 µg/mL of lipopolysaccharide (LPS) for 1 h and then treated with 0.1 mM of DEX or DEX-loaded LPNCs for 24 h.

RNA isolation and RT-PCR: Total RNA was isolated from cultured cells using the SV Total RNA Isolation System (Promega, Madison, WI, USA), following the manufacturer’s instructions. A total of 1 µg of RNA was reverse transcribed to cDNA with M-MLV Reverse Transcriptase (Life Technologies) and random hexamers (Life Technologies). Analyses of the TNFα and GAPDH (endogenous control) mRNA levels were performed by RT-PCR, using commercial TaqMan gene expression assays (Applied Biosystems). Relative quantification of gene expression was assessed using the ΔΔCT method, as described in the TaqMan user manual (User Bulletin no. 2; Applied Biosystems). Gene expression levels for TNFα were normalized to the GAPDH gene. The amounts of mRNA are expressed as arbitrary units.

Statistical analysis: Results from the cell viability and cytokine modulation were statistically analyzed by using GraphPad Prism (8.0.2, 2019, La Jolla, San Diego, CA, USA), with Student’s paired *t* test for cytotoxicity assays and two-way ANOVA for cytokine modulation assays. In all cases, differences were considered significant when the *p*-value < 0.05.

## 3. Results and Discussion

### 3.1. Screening of Experimental Variables on Nanoparticle Properties

The pharmaceutical interest of cellulose derivatives has increased in recent years, due to economic factors (usually due to the low-cost of the excipients, compared with synthetic compounds) and as they originate from renewable resources [24]. EC was selected as the LPNC polymer, as it has been approved by the Food and Drug Administration (FDA) for medical and food applications (i.e., vitamin and mineral tablets), and it has shown good biocompatibility in ocular drug delivery systems [25,26,27]. Furthermore, EC is cheaper than other commonly used polymers, such as PLGA or polycaprolactone. EC is commonly used in the pharmaceutical industry as a coating material for different drug delivery applications, due to its controlled release properties caused by the porous structure, where the drugs can be trapped and diffuse [24].

The choice of organic phase is a parameter to consider, in terms of toxicity. Chloroform and dichloromethane are organic solvents that are commonly used in the Emulsion Solvent Evaporation method; however, due to their toxicity, it is advisable to avoid them when possible. In this case, ethyl acetate and ethanol were selected, as they have been classified as class III (or low toxic potential) solvents by the European Medicines Agency (EMA) [28]. In the Emulsion Solvent Evaporation method, the viscosity of the organic phase influences the diffusion to the aqueous phase. An organic phase that is too viscous will not diffuse adequately, forming large aggregates that cannot be stabilized in the final formulation. This may be attributed to the increase in viscosity of the dispersed phase, resulting in a reduction of the net shear stress and prompting bigger nanodroplets. The ratio between the continuous and dispersed phases is also an important factor: In the solvent evaporation emulsion method, a high proportion of dispersed phase causes an increase in the size of the nanoparticles, due to the increase in droplet size. Furthermore, due to the hydrophobic nature of DEX, the burst effect would increase, and the encapsulation efficiency would decrease. To avoid this problem, dilutions of EC were made in different binary mixtures of ethanol and ethyl acetate and the viscosities of the different organic phases were characterized. The LPNCs were designed with MCT as the oil core, to increase the %EE and reduce the DEX burst release effect. Benzalkonium chloride was incorporated as a preservative.

Table 3 shows the viscosity results for the different binary mixtures of ethyl acetate (EA) and ethanol (ET) with EC at 2% *w*/*w*. The 1:1 and 5:1 ratios were selected as optimal, as they showed the lowest viscosity values. This means a decrease in the viscous forces resisting droplet breakdown and, thus, smaller oil droplets were formed, resulting in a decreased particle size. When LPNCs were produced at the 1:1 ratio, aggregates were observed; so, finally the 5:1 EA:ET ratio was selected as definitive, as it did not show aggregates and obtained low viscosity. To increase the loading capacity of the LPNCs, the EC concentration was increased to the maximum concentration possible to obtain LPNCs with adequate properties, in term of size and PDI [29]. The final EC concentration was fixed at 2.33% *w*/*w* in the organic phase (0.35% *w*/*w* in the final formulation) and the MCT concentration was set at 0.2% *w*/*w*, since at higher concentrations aggregates began to appear.

The stability of LPNCs is directly related to their size, PDI, and Z-potential. Surfactants have an important role in these characteristics and, so, the effect of surfactant concentration on the physicochemical properties of LPNCs was studied. As in any colloidal suspension, lipomers can undergo destabilization processes, such as Ostwald ripening. This process can be avoided by steric stabilization using Tween 80 [30]. Due to its HLB (14.9), Tween 80 acts as a stabilizer of the aqueous phase, coating the surfaces of the LPNCs and preventing their aggregation. Due to the more lipophilic nature of Span 60 (HLB 4.7), it becomes dispersed in the LPNC core when MCT is used [31]. In this case, the percentages of Tween 80 and Span 60 were modified between 1.5% and 2.5% *w*/*w* and 0.16% and 0.32% *w*/*w*, respectively. As it can be seen from Table 4, four batches were produced and the Z-average, PDI, Z-potential, and *%EE* were characterized for each of them.

**Figure 1:**
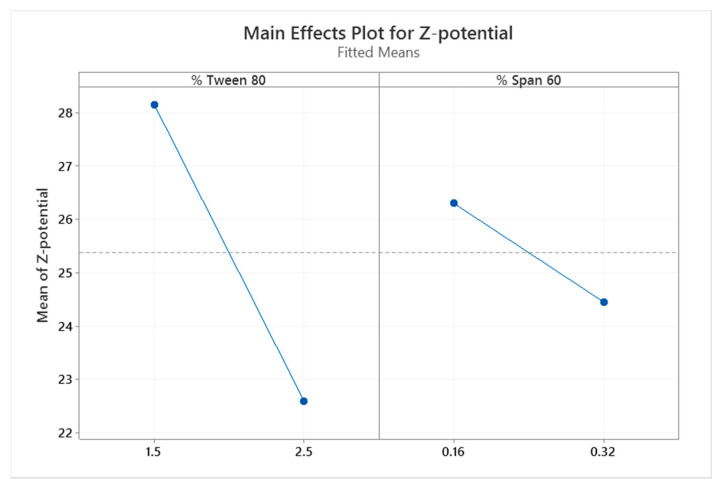
Plots of the main effects of Tween 80 and Span 60 on the Z-potential of the lipid core polymeric nanocapsules (LPNCs).

A general linear model regression using the Minitab software was performed (with a significance level of α = 0.05) to evaluate the effect of the tested experimental variables. The levels of Tween 80 and Span 60 were not significant for the hydrodynamic diameter, the PDI, or for the *%EE*, but they were significant for the Z-potential (*p* = 0.006 and *p* = 0.017, respectively). In Figure 1, the effects of these surfactants on the surface charge of the LPNCs can be observed. By increasing the amount of surfactant, the positive charge was reduced, as the hydroxyl groups of Tween 80 and Span 60 shielded the positive charge of the nanoparticles.

Thus, formulation LP03 was selected as the final formulation, with lower levels of both surfactants (1.5% *w*/*w* of Tween 80 and 0.16% *w*/*w* of Span 60). The %LC of batch LP03 was calculated following Equation (2), which resulted in 30.22%, thus confirming the high loading capacity of this type of nanoencapsulation system [5] compared to other drug delivery systems like liposomes, in which Amin et al. reached an LC of 12.6% [16].

Table 5 shows the residual EA and ET found after the evaporation process. EA has a vapor pressure at 25 °C of 1.23 atm, which is higher than the vapor pressure of ethanol at the same temperature (0.08 atm). Due to the higher volatility of EA than ET, after 5 min of rotary evaporation at 40 °C, the residual quantity was already below the limit of quantification (LOQ). Regarding ethanol, with the mildest evaporation conditions, more residues were found, but they were well below the limit established for residual solvents of class 3 (5000 ppm) in the guideline for residual solvents (ICH Q3C) of the European Medicines Agency. Thus, the evaporation conditions were fixed at 5 min and 40 °C.

### 3.2. Physicochemical Characterization of the LPNCs

**Figure 2:**
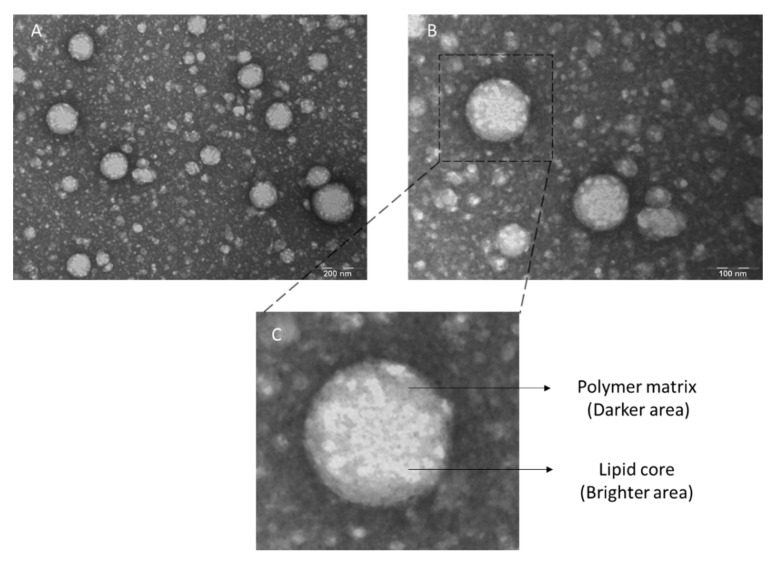
Transmission Electron Microscopy (TEM) images of negative-stained lipid–polymer hybrid LPNCs with 2% uranyl acetate at (**A**) 250,000× magnification; and (**B**) 300,000× magnification. (**C**) Individual nanoparticle representation zoom.

Thanks to the high resolution of the TEM images (Figure 2), it is possible to appreciate the lipid nucleus (brighter area) and distinguish it from the polymeric framework (darker area), as can be observed in Figure 2C. Furthermore, the spherical morphology was confirmed. A total of 180 particles were analyzed and a mean size of 126.40 ± 34.93 nm was obtained.

**Figure 3:**
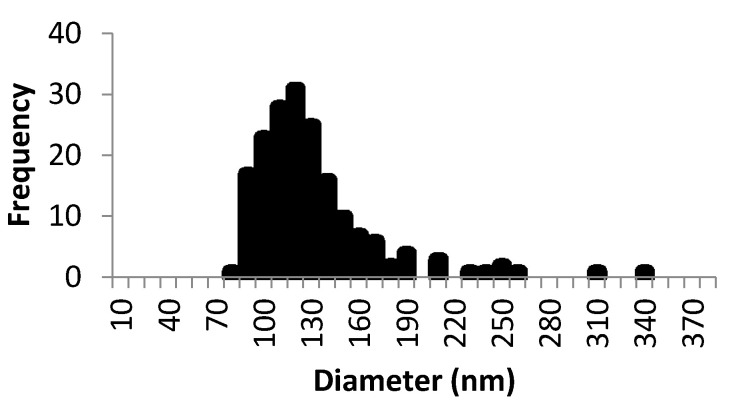
Histogram of lipomers from the measurement of the diameter of the particles (*n* = 180) using ImageJ software.

The diameter distribution of the particles, as measured by TEM, is shown in Figure 3 as a histogram. The average obtained was similar using both TEM and DLS techniques and the %BIAS error for the diameter was 10.78%, which is low.

### 3.3. In Vitro Release Test of DEX-Loaded LPNCs and Free DEX

**Figure 4:**
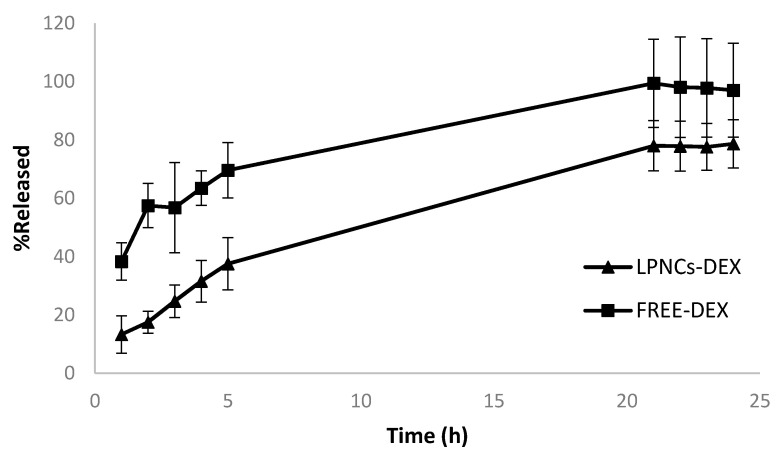
Mean release data obtained from the DEX-loaded LPNCs and from the ethanolic solution of DEX (FREE-DEX) after 24 h of the in vitro release test.

The release data of the free DEX and LPNCs can be seen in Figure 4. After 24 h, the DEX formulated in an ethanolic solution (FREE-DEX) was released at 100%, while the release of DEX loaded in LPNCs (LPNCs-DEX) was more sustained, due to diffusion through the polymeric framework and the affinity of the active for the lipid cores of the particles.

After adjusting the mean release data with the different mathematical models (see Table 6), it was observed that Weibull was the model that best fit (AIC 28.81) the experimental data in the case of LPNCs, and Korsmeyer–Peppas was the model that best fit (AIC 44.86) in the case of DEX formulated in a hydroalcoholic solution.

Once the mean release kinetic behavior was determined, the mean and standard deviation of the individual release data for free DEX and LPNCs-DEX were obtained, as reported in Table 7.

Although the Weibull equation is a non-mechanistic equation, Papadopoulou et al. [22] obtained a relationship between the shape parameter β and the release mechanism, in the same way as the power law equation (Korsmeyer–Peppas equation) does with the exponent n. In the case of LPNCs-DEX, the value of β was between 0.75 and 1, which corresponds to a combined release mechanism; that is, diffusion in a normal Euclidean substrate with the contribution of another release mechanism. This fact was confirmed by the *n* value (0.573) found for LPNCs reported in Table 6, corresponding to anomalous transport or a combined mechanism. Due to the hybrid nature of these nanoparticles, the active ingredient could be distributed both in the lipid core and the polymeric framework, resulting in an overlap of both release kinetics, such that anomalous transport would be observed. In the case of FREE-DEX, this study was below the limits set in the Korsmeyer–Peppas model (i.e., 0.43). However, Singhvi and Singh [32] reported that, although the value of n obtained was not in the range suggested in Korsmeyer–Peppas model, it also indicates a diffusion-controlled drug release mechanism. This fact could be confirmed by the Weibull model, which had an AIC value close to that of Korsmeyer–Peppas, where the β value for FREE-DEX was 0.698 (i.e., between 0.69 and 0.75), which would indicate the release as a normal diffusion mechanism dominated by the concentration difference without the contribution of other release mechanisms, unlike the case of LPNCs.

### 3.4. Confocal Microscopy Biodistribution of Fluorescent Probes

**Figure 5:**
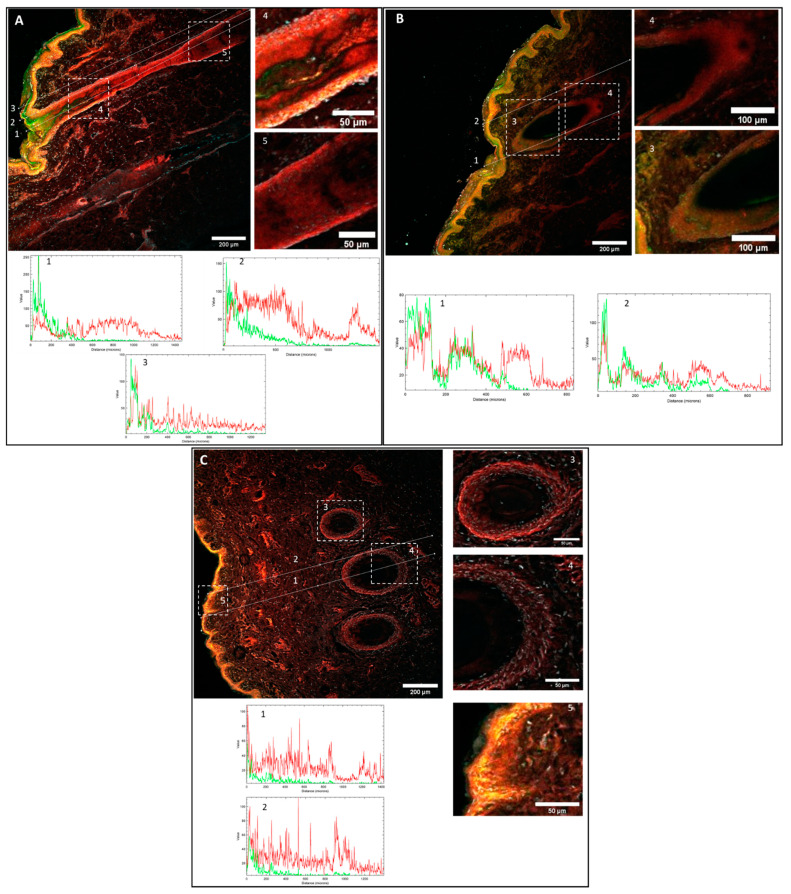
Confocal Microscopy Fluorescence images and plot intensity profiles of pig skin cross-sections. Green color corresponds to C6 and red to LRB fluorescence. Yellow color corresponds to the overlap of C6 and LRB: (**A**,**B**) C6-loaded LRB-labelled LPNCs. (**C**) Free C6 and LRB control solution. Lines numbered 1–3 in Figure 5A; 1–2 in Figure 5B and 1–2 in Figure 5C correspond to Multichannel intensity plot profiles as function of the depth (µm). Dashed squares numbered 4–5 in Figure 5A, 3–4 in Figure 5B and 3–4 in Figure 5C correspond to zoomed in regions. The images were captured using 10× magnification.

After the permeation experiment, skin was observed under a confocal microscope, to evaluate the skin distribution of both fluorochromes. The literature has described a high number of fluorochromes that are included in nanoparticles for localization in the skin. Usually, the selection of the tracking compound is based on the aim of the experiment. In this case, C6 was selected as the drug model and LRD to track the nanocapsules. According to the structure of LRD, the hydrocarbon tail would be inside the lipid core and the rhodamine head would likely be located in the polymer shell or in the interphase lipid–polymer in the nanoparticle structure, as with Span 60. To improve visualization, cell nuclei were stained with Hoechst (blue color). The results obtained from the confocal biodistribution study are shown in Figure 5. The autofluorescence value in the red channel (corresponding to LRD) was 5.9 AU and that of the green channel (corresponding to C6) was 0.3 AU. These values were subtracted from the mean intensity of the samples. Using ImageJ software, linear segments were drawn to analyze the intensity profile as a function of depth (in µm). Yellow color corresponds to the co-localization of both fluorochromes.

Figure 5A,B show the biodistribution patterns of the C6 and LRB fluorophores vehiculated in LPNCs (C6-LRB-LPNCs).

In Figure 5A, three lines are drawn from the stratum corneum to deeper layers of the skin. Line 1 corresponds to the central section of the hair follicle, line 2 corresponds to the follicle edge (outer root sheath area), and line 3 corresponds to a non-follicle section. In the central section (line 1), the intensity of C6 is between 100 and 200 AU in the infundibulum (depth < 100 µm). At depths of 200 µm, the intensity of C6 drops to 50 AU and, as the depth increases, the intensity of the green channel continues to decrease, to about 20 AU at 400 µm (corresponding to the hair follicle isthmus). Beyond 500 µm, in depth (i.e., in the suprabulbar region), the intensity of C6 was low (<10AU), which seems to indicate that C6 does not accumulate in the deeper parts of the follicle. Regarding the intensity in the red color, corresponding to the presence of LRB, a different behavior can be observed. The profile of the center of the follicle (Figure 5A, Line 1) shows an intensity value around 50 AU from the skin surface to 700–800 µm deep. The profile of the border of the follicle (Figure 5A, Line 2) shows a similar profile, with the intensity of the LRB up to 700 µm being around 65 AU. Due to the narrowing of the follicle, the line is outside the follicle, around 1000 µm; however, near the follicle sheath (1200 µm), it can be seen that the intensity of the LRB was around 50 AU. In the non-follicle area (Figure 5A, Line 3), the C6 intensity profile was high to 200 µm in depth, but then decreased quickly, suggesting the accumulation of C6 in the epidermis. It can also be seen that the intensity of LRB outside the follicle at depths greater than 200 µm was clearly lower than that of the follicle lines. In the epidermis, a yellow color can be observed, corresponding to the co-localization of both fluorochromes in this region. In other words, LRB had accumulated throughout the follicle at depths greater than C6.

In Figure 5B, corresponding to another skin section of the permeation with C6-LRB-LPNCs, a section of the hair follicle can be seen. In this case, the section is not longitudinal but a cross-section. The intensity profiles of the different skin annexes and hair follicles followed the same biodistribution for both fluorophores as previously discussed. Accumulation of C6 and LRB was seen up to about 400–500 µm deep. At deeper follicle depths, only LRB accumulation was observed.

Different magnifications were made on sections of the hair follicle, in order to highlight the fluorochrome distribution in the infundibulum (Figure 5A, zoom 4) and in the isthmus area (Figure 5A, zoom 5). In the zooms of Figure 5B, zoom 3 and zoom 4 highlight the transversal hair follicle section with the different fluorochrome distribution previously described.

In Figure 5C, the zoom after permeation of the FREE-C6-LRB control solution can be observed. In this case, the observed biodistribution was different. As they were not delivered in nanoparticles, the fluorophores did not accumulate in the hair follicles. As can be seen in the profiles of lines 1 and 2 of Figure 5C, the intensity of C6 was around 20 AU in the epidermis and decayed at depths greater than 100 µm. The LRB intensity was around 20 AU throughout the entire zoom where you can see the dermis, as well as the hair follicle cross-section.

In addition, when analyzing zooms 3 and 4 of Figure 5C, it can be observed that the intensity of the yellow color in the follicles is almost non-existent, due to the non-accumulation of C6 in the hair follicle. In zoom 5 of Figure 5C, the accumulation of C6 and LRB in the epidermis of a superficial invagination (<100 µm) can be seen.

These results explain the biodistribution of these two hydrophobic fluorophores carried in the LPNCs. The intensity of the green color, corresponding to the accumulation of C6, was greater in the epidermis and in the upper part of the hair follicle, and decreased with increasing depth in the skin. The images obtained by confocal microscopy seem to indicate that the in vitro skin penetration by C6 in the LPNCs accumulated on the epidermis and the hair follicle infundibulum, but did not penetrate into the deepest parts of hair follicles. This fact suggests that C6 is released when it comes into contact with the stratum corneum lipids, due to its hydrophobic nature, leading to greater accumulation in less deep areas.

The intensity in red color, corresponding to LRB, was less than that of C6 in the epidermis but greater than that of C6 when analyzing the profile deeper into the hair follicle. The fact that an accumulation of LRB was observed at these depths suggests that this fluorophore is found in the lipid nucleus of the nanoparticles and travels with them towards the bulb of the follicle. As described in the literature, ethyl cellulose polymer nanoparticles accumulate in skin annexes, due to their geometry and size, and can deliver small molecules to hair follicles through the transfollicular route, limiting distribution to the rest of the skin and to systemic circulation [33].

### 3.5. Immunohistofluorescence Biodistribution

Confocal laser microscopy is usually employed to study the biodistribution of nanoparticles in different tissues; unfortunately, this is an indirect measurement, as there is no direct tracking of the API. There exist other techniques to study topical biodistribution, such as skin layer separation [34], cyanoacrylate tape-stripping [35], or dermatopharmacokinetics [36]. Techniques such as cyanoacrylate-stripping allow to study hair follicle targeting but usually have a lower spatial resolution than IHF and could have problems, such as analytical interferences, quantification limit issues, lack of complete recovery, or matrix effects. By means of the IHF, it is possible to visualize if there is accumulation in the different anatomical regions of the hair follicle.

Due to this and to confirm the data obtained in the previous section, an IHF experiment was carried out. Rabbit anti-DEX IgG and Alexa Fluor 488 goat anti-rabbit were employed, such that direct visualization of the drug could be performed.

**Figure 6:**
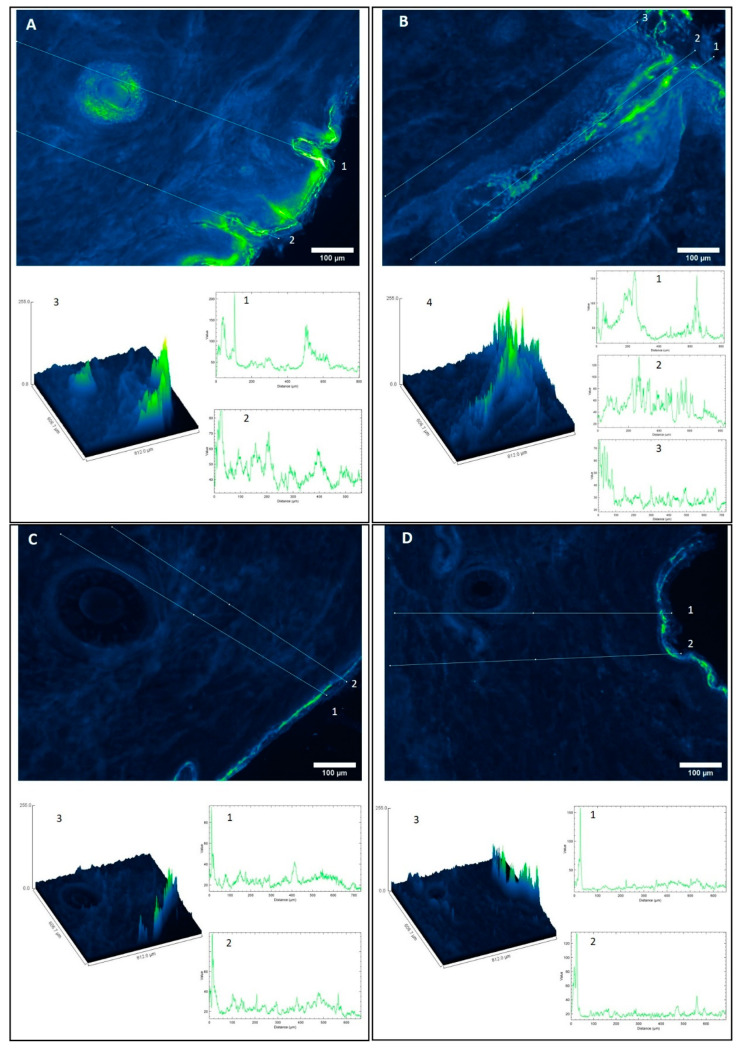
IHF images of a pig skin cross-section. Green color corresponds to high intensity and blue to lower intensity fluorescence of Alexa 488 secondary antibody (Ab)–Primary Ab–DEX complex: (**A**,**B**) DEX-loaded LPNCs; (**C**,**D**) hydroalcoholic DEX control solution. Figure 6A 1–2, Figure 6B 1–3, Figure 6C 1–2 and Figure 6D 1–2 correspond to green intensity plot profiles as function of the depth (µm). Figure 6A 3, Figure 6B 4, Figure 6C 3 and Figure 6D 3 correspond to surface plots. The images were captured using 10× magnifications and the Lookup Table (LUT) used to show the pictures was Green Fire Blue.

After the IHQ experiment, a different behavior was observed, with respect to the DEX-FREE (Figure 6C,D) and LPNCs-vehiculated DEX (Figure 6A,B). The visualization filter used in ImageJ was Green Fire Blue, where the blue color corresponds to the lowest intensity and the yellow-green color to the highest intensity. Figure 6B shows a longitudinal section of the hair follicle. In panels A, C, and D of Figure 6, cross-sections of the hair follicles are observed. Surface plots were obtained, in order to compare the intensity in a more visual way.

In the same way as in Section 3.6, different lines were drawn to compare the intensity profiles. Line 1 in Figure 6A corresponds to the intensity across the lateral zone of a hair follicle. In this case, an accumulation of DEX was seen (intensity around 140 AU at 500 µM depth). Line 2 corresponds to permeation outside the follicle, for which it was observed that DEX remained restricted to the epidermis and its invaginations, with an intensity of 60 AU. In Figure 6B, three lines are drawn. Lines 1 and 2 correspond to the central zone and the outer sheath of the follicle, respectively. Although the maximum intensity (approximately 100 AU) was observed at around 250 µm, DEX accumulation went to 700 µm, corresponding to the hair follicle bulb (Figure 6B, Zoom 1 and 2). Line 3 corresponds to an area outside the follicle; in this case, the intensity dropped to 25 AU when entering beyond 100 µm and, again, it was observed that DEX remained restricted to the area of the epidermis and stratum corneum. The intensity along the follicle remained around 40 AU—almost double compared to outside the follicle. Surface plots of both images were also made, to obtain a visual image of the accumulation of DEX. It is clear that accumulation in hair follicles has occurred.

Regarding the permeation of FREE-DEX, the behavior was different. Lines 1 and 2 of Figure 6C,D are very similar and show that DEX did not accumulate in hair follicles when it was formulated in this solution. Beyond 50 µm in depth (epidermis), the intensity was low (about 20 AU). This behavior is shown in the surface plots in Figure 6C Zoom 3 and Figure 6D Zoom 3, where FREE-DEX did not accumulate in follicles, only in the stratum corneum and part of the epidermis.

When comparing these results with those obtained by confocal microscopy, it can be observed that the biodistribution of DEX was similar to that of LRB, as it accumulated in the deep areas of the follicles (>400 µm). These results corroborate the high encapsulation efficiency results and suggest that it is possible to achieve a controlled and sustained release in the hair follicle by encapsulation in LPNCs. As a result, a depot effect in the pilosebaceous unit is expected. This effect could, on the one hand, reduce the frequency of administration (from the 12 h usually used in topical corticosteroids to 24 h) and then could reduce the associated adverse effects. On the other hand, a depot effect and the release of the drug in the site of action would improve the treatment efficacy and reduce or avoid the systemic or intralesional administration of the drug, reducing the systemic adverse effects (which are usually worse than the adverse effects of topical administration) and patient discomfort caused by injections (in the intralesional therapy). These facts should be evaluated in future clinical trials.

### 3.6. Cytotoxicity and Anti-TNFα Efficacy

**Figure 7:**
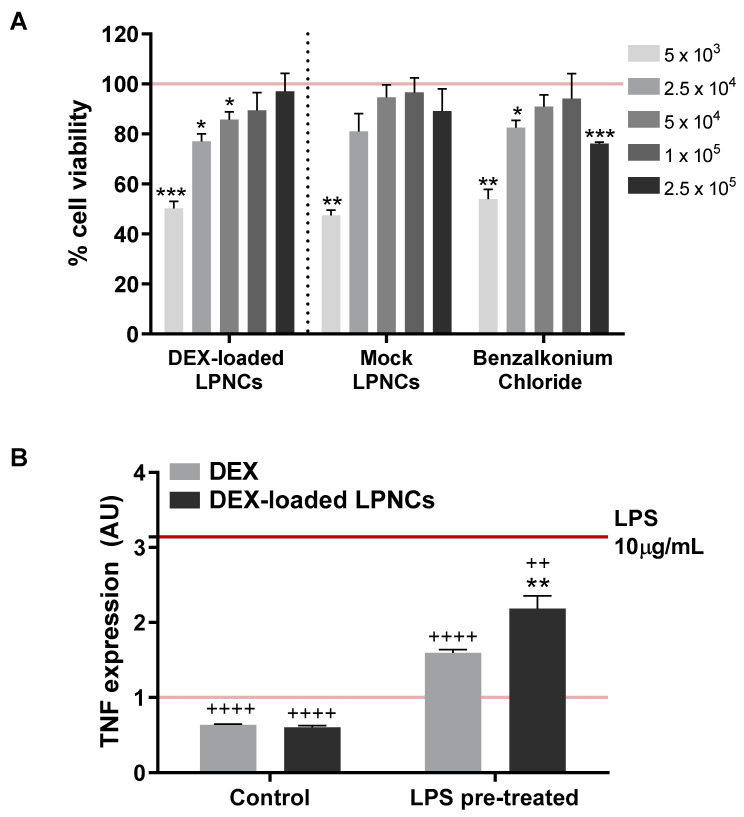
In vitro cell culture studies: (**A**) HEK001 cell viability with MTT assay. The indicated numbers represent the inverse dilution factors, referring to the composition of synthetized LPNCs. For DEX-loaded LPNCs, the dilution factors correspond to the concentrations of 5, 1, 0.5, 0.25, and 0.1 µM of DEX. For benzalkonium chloride, proper concentrations were 1, 0.2, 0.1, 0.05, and 0.02 µM. Data are represented as the mean ± SEM (*n* = 3) of the cell viability percentage, referring to untreated controls (horizontal lane). Statistical significance was assessed by Student’s paired t-test; * *p* < 0.05; ** *p* < 0.01; *** *p* < 0.005. (**B**) TNFα mRNA expression was measured after treating cells for 24 h with 0.1 µM of either free dexamethasone (grey) or DEX-loaded LPNCs (dark gray), without (left) or with (right) a 1-h pre-treatment with LPS (10 µg/mL). TNFα expression is represented as the mean ± SEM (*n* = 3); TNFα expression at control and LPS treatment conditions is indicated with horizontal pink and red lanes, respectively. Statistical significance was evaluated by one-way ANOVA compared to the control (*) or LPS (+); ** *p* < 0.01, ++ *p* < 0.01, ++++ *p* < 0.001.

Human-transformed keratinocytes (HEK001 cells) were chosen to assess the DEX-LPNC cytotoxicity (Figure 7A). Non-loaded LPNCs, benzalkonium chloride, and untreated cells were tested as controls. For loaded LPNCs, the inverse dilution factors (5000, 25,000, 50,000, 100,000, and 250,000) corresponded to the respective concentrations of 5, 1, 0.5, 0.25, and 0.1 µM of DEX. For benzalkonium chloride, proper concentrations were 1, 0.2, 0.1, 0.05, and 0.02 µM. The cell viability profiles of the tested compounds exhibited a similar pattern: there was a viability reduction, compared to untreated cells, at higher concentrations of loaded and non-loaded LPNCs. To ascertain the reason for this toxicity, pure benzalkonium chloride was evaluated with the same dilution scheme as the LPNCs, demonstrating that the reason for the viability reduction was caused by the preservative. Although this fact was observed, there exist different commercial products that use the same preservative for the topical route, as described in the FDA inactive ingredients data base [37], demonstrating the suitability of the selected preservative system.

To evaluate the in vitro anti-inflammatory efficacy of the LPNCs, TNFα was selected as a tracker, as it is usually involved in most inflammatory alterations of the skin, particularly in AA [38]. Furthermore, in many cases, it has been identified as a promising target for pharmacological modulation [39]. Figure 7B shows the TNFα expression in HEK001 cells after treatment with DEX-LPNCs and FREE-DEX for 24 h after pre-treatment with 10 µg/mL of LPS to induce inflammation. LPNCs were tested at a DEX level equivalent to 0.1 µM, as the cell viability was not reduced at this concentration level. FREE-DEX at the same concentration level was used as control. A significant reduction in TNFα can be seen in both cases. The lower efficacy of the lipomers, compared with the solution, could be caused by the differential release pattern observed in Figure 4: at 24 h, the amount of DEX released from the lipomers was around 75%, compared with 100% in the solution. Similarly, the difference between the FREE-DEX and lipomers was about 27%, such that the drug release limited the anti-TNFα efficacy. This difference is not expected to appear in real applications, considering that the LPNCs have accumulated in hair follicles and, so, a depot effect would appear (not evaluated in cell culture), such that the slow degradation of the lipomers in the follicle would release the drug into the surrounding and deeper area. After multiple administrations, an increased exposition of DEX would take place, compared with the free drug.

## 4. Conclusions

LPNCs with a high carrying capacity were successfully developed for the active dexamethasone. When studying the biodistribution of the nanoparticles using confocal microscopy, accumulation in hair follicles and cutaneous annexes was observed, thus proving their ability to achieve follicular targeting. These results were confirmed for DEX biodistribution by immunofluorescence, where DEX-LPNCs demonstrated an increase in accumulation in hair follicles, compared to FREE-DEX. The cytotoxicity of the particles was studied, where toxicity (caused by the preservative, benzalkonium chloride) was observed only at high doses. The anti-inflammatory efficacy of DEX-LPNCs, with TNFα as a tracker, was demonstrated. Its high %LC and %EE, good physicochemical properties (size of 115 nm, low polydispersity, and Z-potential of +30 mV), sustained in vitro release profile, localized release in hair follicles, and anti-inflammatory efficacy make this nanoformulation a very interesting candidate to improve the efficacy and reduce the adverse effects of corticosteroids for diseases in which there is inflammation in the hair follicles, such as alopecia areata. Thanks to the follicular targeting obtained, it could be possible to have a depot effect within the pilosebaceous unit, which could allow us to reduce the frequency of administration compared to classical formulations. The safety and efficacy profiles of the DEX-lipomers should be verified in clinical trials to compare side effects.

## Figures and Tables

**Table 1:** Levels studied for the surfactants.

Factor	Lower Level	Higher Level
% Tween 80 (*w*/*w*)	1.5	2.5
% Span 60 (*w*/*w*)	0.16	0.32

**Table 2:** Different kinetic models and equations tested.

Kinetic Model	Equation
First Order	$F=F_{max}\;\left(1-e^{\left(-K_{1}t\right)}\right)$
Higuchi	$F=K_{H}\;\cdot \;t^{1/2}$
Korsmeyer–Peppas	$F=K_{KP}\;\cdot \;t^{n}$
Weibull	F=1−e(−tTd)β

**Table 3:** Organic phase ethyl cellulose (EC) mixture viscosities.

EA:ET Ratio	Viscosity (cP)
1:1	16
5:1	20.5
1:5	24
1:0	70.5
0:1	25

**Table 4:** Influence of Span 60 and Tween 80 on the physicochemical parameters.

Batch	% Tween 80	% Span 60	Z-Average (nm)	PDI	Z-Potential (mV)	% EE
LP01	2.5	0.32	117.6 ± 1.2	0.263 ± 0.005	21.7	96.76
LP02	1.5	0.32	130.5 ± 1.1	0.215 ± 0.011	27.2	96.68
LP03	1.5	0.16	114.1 ± 1.1	0.239 ± 0.002	29.1	96.93
LP04	2.5	0.16	125.9 ± 0.5	0.256 ± 0.006	23.5	98.32

**Table 5:** Residual solvents after evaporation at 40 °C.

Evaporation Time (min)	Residual EA (ppm)	Residual ET (ppm)
5	<LOQ	153.0 ± 10.0
7	<LOQ	34.4 ± 4.5
10	<LOQ	12.5 ± 2.3
15	<LOQ	1.5 ± 0.0

**Table 6:** Model selection and parameter estimation of the LPNCs-DEX and FREE-DEX. Bold type indicates the selected model, based on the lowest AIC.

Formulation	Model	AIC	Parameters	Value
LPNCs-DEX	First order	31.84	k (h^−1^)	0.128
	Higuchi	39.98	k_H_ (%h^−1/2^)	16.263
	Korsmeyer–Peppas	39.13	k_KP_ (%h^−n^) n	7.91 0.573
	**Weibull**	**28.81**	**T_d_ (h)** **β**	**13.49** **0.79**
FREE-DEX	First-order	68.51	k (h^−1^) Fmax (%)	0.192 104.35
	Higuchi	71.60	k_H_ (%h^−1/2^)	22.475
	**Korsmeyer–Peppas**	**44.86**	**k_KP_ (%h^−n^)** **n**	**42.78** **0.271**
	Weibull	47.43	β Td (h)	0.698 3.21

**Table 7:** Individual modelling and parameters for the LPNCs and FREE DEX release data.

Formulation	Model	Parameters	Value
LPNCs-DEX	Weibull	β T_d_ (h)	0.82 ± 0.17 14.16 ± 4.11
FREE-DEX	Korsmeyer–Peppas	k_KP_ (%h^−n^) n	42.39 ± 5.96 0.278 ± 0.034

## Data Availability

The data presented in this study are available on request from the corresponding author. The data are not publicly available due to intellectual property.
